# 

**Published:** 1883-09

**Authors:** 


CONTENTS.

| | Page. | | Page. |
|---|---|---|---|
| Warnings | 185 | Quinine in the Treatment of Whooping Cough | 196 |
| Neuralgia | 187 | The Planet Mars | 197 |
| Rheumatism | 188 | The Home Doctor | 198 |
| White Decay | 189 | Annie Laurie | 198 |
| The Prince's Escape | 190 | How a Man Walks | 198 |
| Treatment of Goitre | 192 | St. Vitus' Dance | 200 |
| After-Dinner Speeches of Americans | 193 | Food Adulteration | 202 |
| Made Useful | 194 | The Egyptian Sphynx | 203 |
| Ice for Teething Children | 195 | | |

